# Development of new bioactive molecules to treat breast and lung cancer with natural myricetin and its derivatives: A computational and SAR approach

**DOI:** 10.3389/fcimb.2022.952297

**Published:** 2022-09-27

**Authors:** Shopnil Akash, Ajoy Kumer, Md. Mominur Rahman, Talha Bin Emran, Rohit Sharma, Rajeev K. Singla, Fahad A. Alhumaydhi, Mayeen Uddin Khandaker, Moon Nyeo Park, Abubakr M. Idris, Polrat Wilairatana, Bonglee Kim

**Affiliations:** ^1^ Department of Pharmacy, Faculty of Allied Health Sciences, Daffodil International University, Dhaka, Bangladesh; ^2^ Laboratory of Computational Research for Drug Design and Material Science, Department of Chemistry, European University of Bangladesh, Dhaka, Bangladesh; ^3^ Department of Pharmacy, BGC Trust University Bangladesh, Chittagong, Bangladesh; ^4^ Department of Rasa Shastra and Bhaishajya Kalpana, Faculty of Ayurveda, Institute of Medical Sciences, Banaras Hindu University, Varanasi, Uttar Pradesh, India; ^5^ Institutes for Systems Genetics, Frontiers Science Center for Disease-Related Molecular Network, West China Hospital, Sichuan University, Chengdu, China; ^6^ School of Pharmaceutical Sciences, Lovely Professional University, Phagwara, India; ^7^ Department of Medical Laboratories, College of Applied Medical Sciences, Qassim University, Buraydah, Saudi Arabia; ^8^ Centre for Applied Physics and Radiation Technologies, School of Engineering and Technology, Sunway University, Bandar Sunway, Selangor, Malaysia; ^9^ Department of Korean Medicine, Kyung Hee University, Seoul, South Korea; ^10^ Department of Chemistry, College of Science, King Khalid University, Abha, Saudi Arabia; ^11^ Research Center for Advanced Materials Science (RCAMS), King Khalid University, Abha, Saudi Arabia; ^12^ Department of Clinical Tropical Medicine, Faculty of Tropical Medicine, Mahidol University, Bangkok, Thailand

**Keywords:** drug design, virtual screening, molecular modeling, breast and lung cancer, molecular docking

## Abstract

Each biopharmaceutical research and new drug development investigation is targeted at discovering novel and potent medications for managing specific ailments. Thus, to discover and develop new potent medications, it should be performed sequentially or step by step. This is because drug development is a lengthy and risky work that requires significant money, resources, and labor. Breast and lung cancer contributes to the death of millions of people throughout the world each year, according to the report of the World Health Organization, and has been a public threat worldwide, although the global medical sector is developed and updated day by day. However, no proper treatment has been found until now. Therefore, this research has been conducted to find a new bioactive molecule to treat breast and lung cancer—such as natural myricetin and its derivatives—by using the latest and most authentic computer-aided drug-design approaches. At the beginning of this study, the biological pass prediction spectrum was calculated to select the target protein. It is noted that the probability of active (Pa) score is better in the antineoplastic (Pa: 0.788–0.938) in comparison with antiviral (Pa: 0.236–0.343), antibacterial (Pa: 0.274–0.421), and antifungal (Pa: 0.226–0.508). Thus, cancerous proteins, such as in breast and lung cancer, were picked up, and the computational investigation was continued. Furthermore, the docking score was found to be -7.3 to -10.4 kcal/mol for breast cancer (standard epirubicin hydrochloride, -8.3 kcal/mol), whereas for lung cancer, the score was -8.2 to -9.6 kcal/mol (standard carboplatin, -5.5 kcal/mol). The docking score is the primary concern, revealing that myricetin derivatives have better docking scores than standard chemotherapeutic agents epirubicin hydrochloride and carboplatin. Finally, drug-likeness, ADME, and toxicity prediction were fulfilled in this investigation, and it is noted that all the derivatives were highly soluble in a water medium, whereas they were totally free from AMES toxicity, hepatotoxicity, and skin sensitization, excluding only ligands 1 and 7. Thus, we proposed that the natural myricetin derivatives could be a better inhibitor for treating breast and lung cancer.

## Introduction

Cancer has the second highest incidence of death in the United States and is a significant public health concern globally because of delays in management, treatment, and diagnosis as a consequence of healthcare facility cutbacks due to the concern that COVID-19 contamination may lead to a rapid decline in cancer deaths, accompanied by a rise in advanced-stage illness and, eventually, higher mortality due to the decrease in or lack of affordable healthcare (Aovi et al; Islam et al., 2021; Yabroff et al., 2021). According to the World Health Organization’s annual statement, 9.6 million patients died in 2018, or one in six fatalities, due to different types of cancer (Akash, 2021; Ferlay et al., 2019). Although numerous types of cancer have been discovered, men are more likely to have cancer of the lungs, prostate, stomach, and liver, whereas women are more likely to develop cancer of the breast, lungs, and thyroid (Akash, 2021; Rahib et al., 2014). The occurrence of and deaths due to cancer are increasing at an alarming rate throughout the continent. It is not easy to pinpoint the exact causes for this increase. Still it is likely related to the aged and demographic expansion, and alterations in the epidemiology are significant risk factors for cancer (Sung et al., 2021).

**Figure 1:**
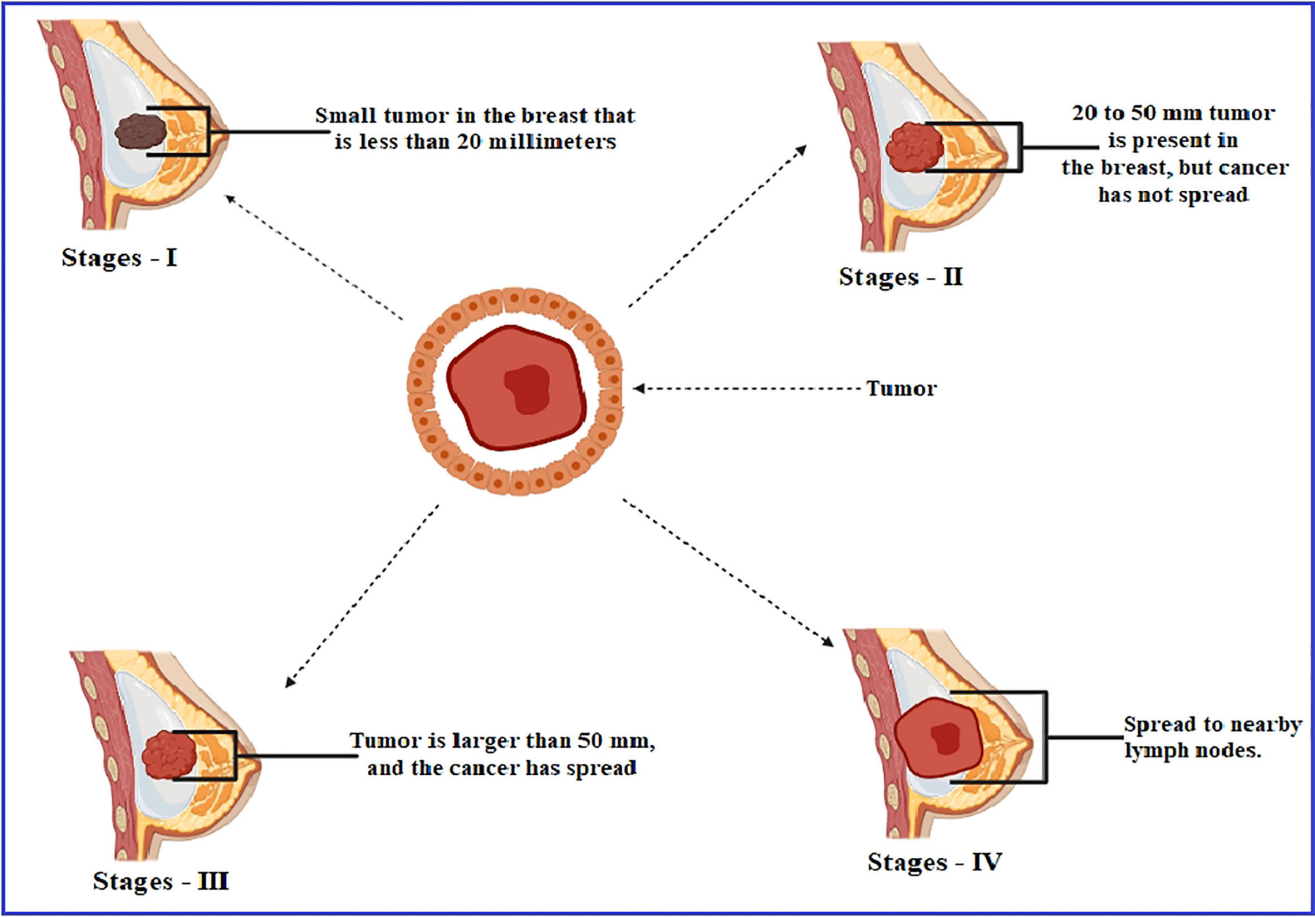
Development stages of breast cancer.

Breast cancer is the most frequent disease affecting women across the globe, contributing to 23% of the 1.1 million new cases of cancer identified in women every year worldwide (Akash and Jahan, 2021; Dange et al., 2017). According to the estimation of the American Cancer Society, 252,710 women in the United States were diagnosed with metastatic breast cancer in 2017, with 40,610 fatalities, which was the second leading cause of death by cancer mortality, behind lung cancer, among women (Jayashree and Velraj, 2017). A study reported that women aged 40–60 comprised 75% of the occurrences, while those aged 60 and above included just 20% of the cases, and female patients younger than 30 were only 5% of the breast cancer circumstances. This report noted that the maximum occurrence of breast cancer is found between the ages of 40 and 60 (Majid et al., 2017). In the majority of the cases, breast cancer arises from tissues in the milk duct membrane and the lobules that feed the ducts with milk (Sudhakar et al., 2013). Genes cause approximately 5–10% of instances passed down from a person’s parents, such as BRCA1 and BRCA2, among other things (Francis et al., 2018). The hER*a* protein, which is found on the surface of tumors, is capable of binding estrogen (17b-estradiol, E2) and promoting cancer cell proliferation and dissemination (Sanchez et al., 2002). The estrogen hormone has been recognized as a significant promoter in forming HE hERa-positive breast cancer, which accounts for about 70–80% of all breast cancers globally (Anderson et al., 2002). The formation of tumors characterizes breast cancer as a result of estrogen signaling after the coupling of estrogen to estrogen receptors (hERa receptors); this amplification of hormone-responsive genes accelerates DNA synthesis and cell replication (Platet et al., 2004). The estrogen-binding receptor is a nuclear receptor, and these binding receptors have fundamental and essential structural features, which are referred to as domains, including the N-terminal (A/B) domain, the DNA-binding domain (C), the hinge domain (D), and the ligand-binding domain (E/F) or the C-terminal domain. This transcription factor interacts with activation function 2 (AF-2), which may be recognized in the domain of ligand binding (Arnal et al., 2013; Shtaiwi et al., 2019) (**Figure 1**).

**Figure 2:**
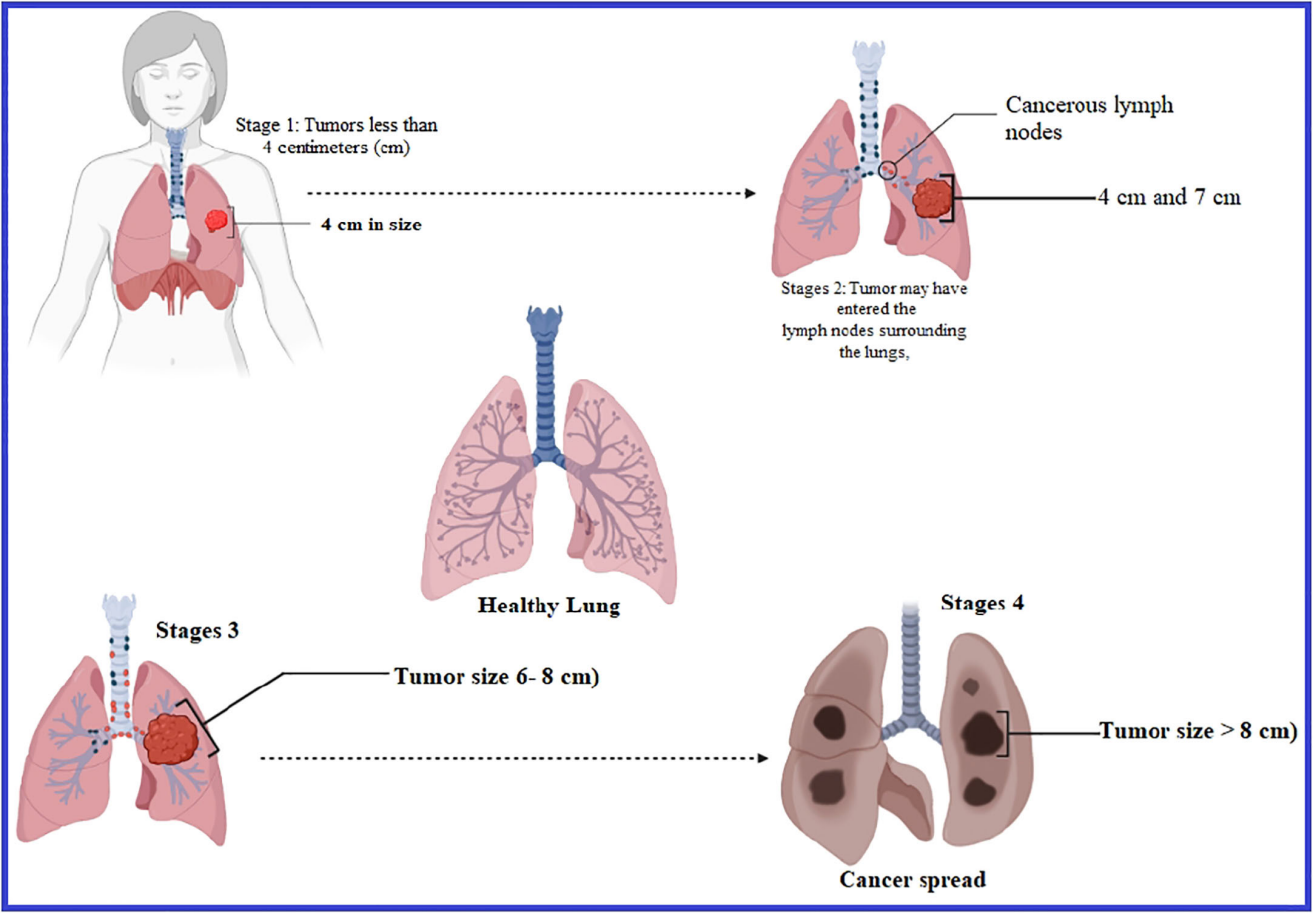
Development stages of lung cancer.

Lung cancer is another problem and the most frequent type of cancer responsible in terms of incidence, which leads to most deaths in male patients all over the world, whereas it is considered the second most significant type of cancer responsible for mortality in women globally (Jemal et al., 2010). On average, nearly 350 people will lose their lives each day to lung cancer in the United States in 2022. According to the projections, lung cancer kills more people than breast, prostate, and pancreatic cancers altogether and is also 2.5 times greater than colorectal cancer (Siegel et al., 2022). It is thought that 81% of the 130,180 lung cancer deaths expected in 2022 will be attributable to tobacco consumption, with an estimated 3,650 fatalities resulting from second-hand smoke (Islami et al., 2018; Rahman et al., 2022; Siegel et al., 2022). Over the last 40 years, there has been no significant progress in developing innovative medicines for lung cancer treatment in its early and metastatic phases. Even though various therapeutic options are available to manage lung cancer in its early stages, lower effectiveness negatively impacts healthy cells and reduces the patients’ survival. Generally, patients with lung cancer are now being treated with surgery, radiation therapy, and chemotherapy among other methods (Hirsch et al., 2017; Huang et al., 2017), but they have shown partial effectiveness and cannot cure fully (**Figure 2**).

Therefore, effective and potential drugs to treat lung and breast cancer are urgently required to reduce deaths through cancer. However, despite the enormous progress in cancer treatment over the last two millennia, cancer patients still face difficulties owing to the poor effectiveness and adverse effects associated with traditional medicines (Bukowski et al., 2020; Wang et al., 2021). Numerous strategies, such as increased drug efflux, genetic mutations, tumor microenvironment, certain growth factors, and an increment in the metabolic rate of xenobiotics, have been implicated in multidrug resistance in cancer, which results in the decrease in the effective frequency of therapies and the slowing down of the opportunity of discovery and development of a targeted and specified remedy (Aldeghaither et al., 2019; Vasan et al., 2019). Additionally, cancer radiation therapy shows its effectiveness by primarily targeting to destroy tumor cells; however, it sparsely impacts the adjoining healthy cells, producing different types of adverse symptoms such as skin peeling, blister formation, itching, and even hair loss (Allen et al., 2017). Consequently, finding an effective medication for cancer therapy is necessary based on a thorough comprehension of the disorder and the limitations of current therapeutic options (Tay et al., 2021). This approach should be focused on the precise target cells while minimizing the risk of destroying the surrounding healthy cells.

Myricetin is a flavonoid from plants widely used in nutritional supplements due to its beneficial properties (Aldeghaither et al., 2019; Vasan et al., 2019). It is one of the most fundamental components found in a wide range of foods and beverages (Allen et al., 2017). This bioactive molecule is also composed of broad-spectrum biological actions, including potent antioxidant (Tay et al., 2021), anticancer (Rahman et al., 2012), antidiabetic (Lagunin et al., 2000), and anti-inflammatory properties (Pires et al., 2015; Aldeghaither et al., 2019). Since myricetin has potential bioactivities that were previously reported at different times, this compound has been picked up and modified by adding or substituting different functional groups. In this research, myricetin has been proposed to develop an effective and potential anticancer medication with fewer side effects.

The *in silico* approaches for early evaluation are appealing because they have the potential to improve the success rates while also lowering the expenses of research and development. Drug research and development spends millions of dollars and requires more than 10–15 years, and only a few medications achieve the effectiveness and safety regulations for FDA clearance after undergoing extensive experimental investigation (Rahman et al., 2012). Thus, in this research, we suggested myricetin and its nine derivatives as an enhanced computational strategy for discovering new potential medications for treating breast and lung cancer.

## Materials and methods

### PASS prediction

The online tool PASS (webpage: http://www.way2drug.com/passonline/) has been introduced to measure the pass prediction. This website intends to predict the physiological and biochemical profiles of organic molecules depending on their structural formulae for more than 4,000 categories of physiological and biochemical activity, with an accuracy of more than 95% for drug-like molecules. It is represented by Pa and Pi values. Normally, the Pa and Pi values are at a maximum of 1, and Pa is not equal to Pi (Lagunin et al., 2000).

### Preparation of ligand and molecular optimization

The Hypercom 8.0 package was used to draw the chemical structures of myricetin and its derivatives, which were then pre-optimized by implementing the molecular mechanics force field (MM+, AMBER) approaches. The molecular structures were subjected to semi-empirical AM-1 to find the homologs with the minimum energy. Finally, the optimized chemical structures were stored in PDB format for further analysis, such as molecular docking, pass prediction, drug-likeness, and ADMET.

### Determination of the data of ADMET

ADMET projection is considered the essential investigation of the development and discovery of new drug molecules. The ADMET qualities are the most valuable for measuring the features and *in silico* pharmacokinetic parameters of the envisaged substances. As a result, it allows for quick and provisional testing of ADMET qualities before the compounds are entirely inspected in vitro since these newly synthesized compounds may be poisonous or produce an adverse effect on the body. Thus, the online web tool pkCSM (http://biosig.unimelb.edu.au/pkCSM/prediction) has been used to project ADMET data (Pires et al., 2015). These ADMET data are calculated and measured based on the chemical properties and structures of molecules.

### Determination of Lipinski rule

The Lipinski rule is another vital parameter in computer-based drug design. The “rule of five” is used to estimate the extent of absorption or permeability of chemicals when they come into contact with the lipid bilayers in the body. These characteristics are evaluated using Lipinski’s “rule of five”, according to which a chemical fits the drug-like criteria if it matches all five of the following criteria (Santos et al., 2016; Kumer et al., 2021):
The molecular weight is less than 500 g/mol.
The calculated octanol/water partition coefficient is less than 5 (logP 5).
The number of hydrogen bond donors is slightly less than 5.
The number of hydrogen bond acceptors (particularly N and O atoms) is not more than 10.

### Protein preparation and molecular docking

**Figure 3:**
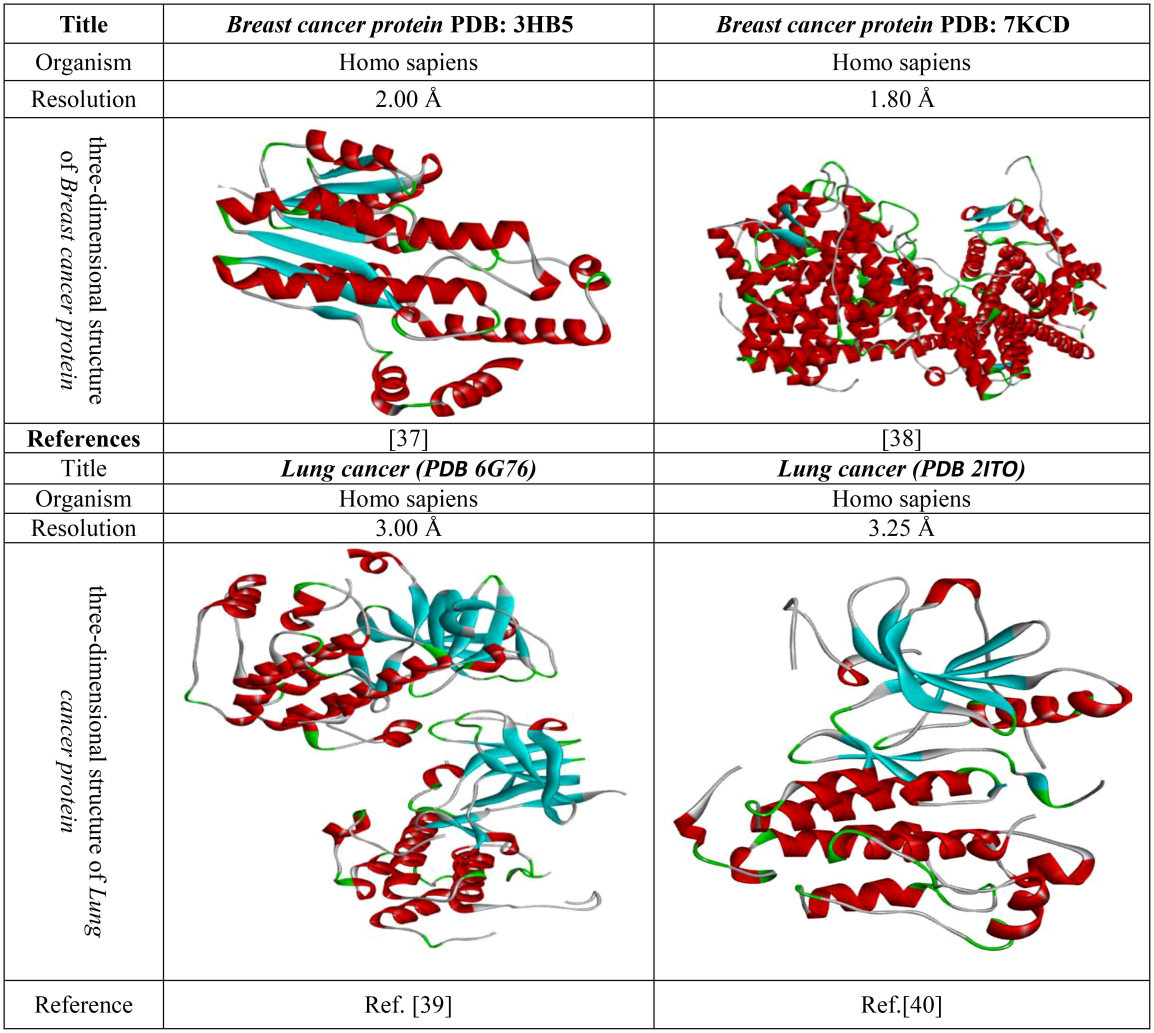
Three-dimensional protein structure of breast and lung cancer.

The use of molecular docking is one of the most influential and appropriate ways of determining drug interactions with proteins. Thus, before the docking analysis, fresh protein should be generated. Firstly, the crystal structure of the 3D protein was downloaded from online resources (PDB or protein databank (Protein data bank, 1971), https://www.rcsb.org/). Then, this protein was uploaded to the Pymol 2020 application, and all the excess and unwanted substances, such as drugs or heteroatoms, were minimized. Then, it was saved in PDB format and uploaded to PyRx software for molecular docking with ligands. The crystal protein structure and their related document are given in **Figure 3**.

## Result and discussion

### Structure–activity relationship

**Figure 4:**
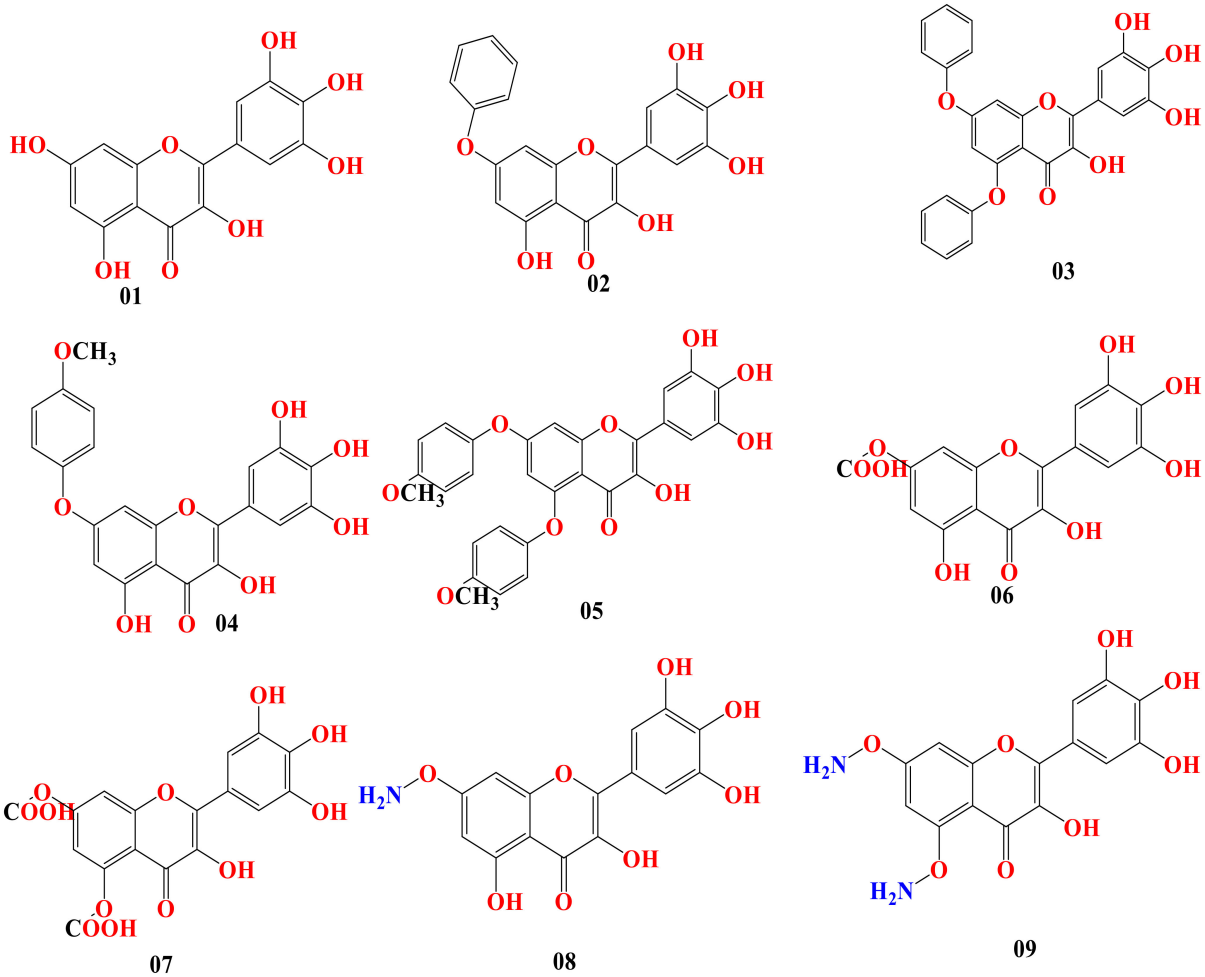
Chemical structure of myricetin and its derivatives.

Predicting the desired bioactivity from the molecular structure is possible using the structure–activity relationship (SAR). It is correlated between drugs and the design of chemicals. This sophisticated technology is utilized in drug development to assist in obtaining or the biosynthesis of attractive novel molecules and to better characterize current medicinal compounds. In our research, myricetin was the primary molecule. At different points of myricetin, we substituted potent functional groups to check the potency against breast cancer and lung cancer. The chemical structure is presented in **Figure 4**.

### Optimized structure of the tested ligand

**Figure 5:**
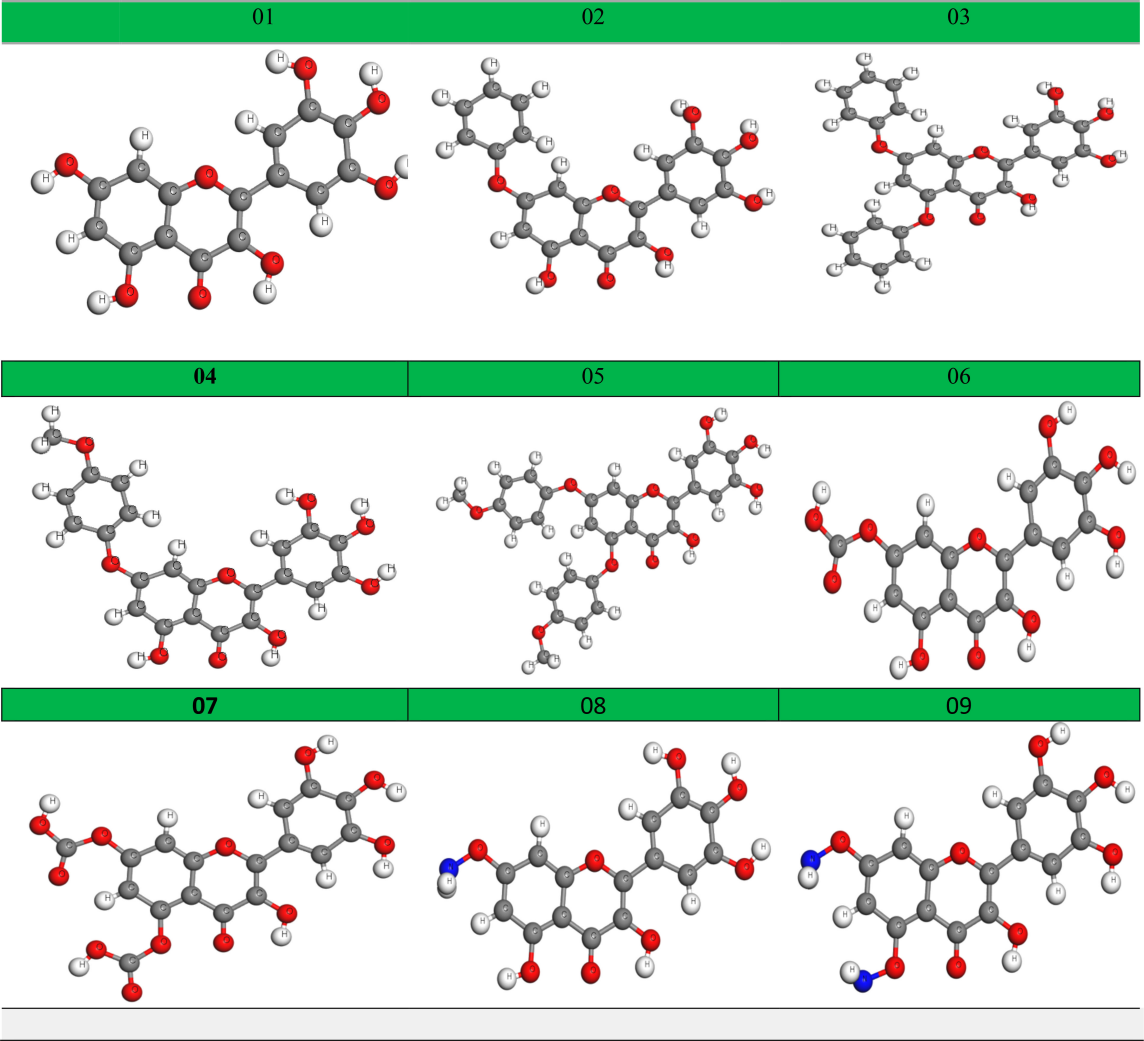
Optimized molecular structure.

Geometry optimization is achieved by altering the atoms of a molecule in order to obtain the most stable shape with the minimum attainable ground-state energy (Chrysostomou et al., 2021). This approach is most prevalent in computational chemistry. Before performing molecular docking, all the tested ligands should be optimized to get the precise results. The optimized chemical figures are drawn and presented in **Figure 5**.

### PASS prediction spectrum

The online-based web tool PASS (prediction of activity spectra for molecules; http://www.pharmaexpert.ru/PASSonline/index.php) was utilized to acquire data on the antiviral, antibacterial, antifungal, and antineoplastic effectiveness of the molecules as developed by SAR studies. The acquisition pass spectrum values obtained, with Pa scores of 0.236–0.343 for antiviral, 0.274–0.421 for antibacterial, 0.226–0.508 for antifungal, and 0.788–0.938 for antineoplastic, were investigated. Here the probability of being active or a greater Pa score can be seen for antineoplastic. Therefore, lung and breast cancers are selected as targeted diseases, and other related studies were performed (**Table 1**).

**Table 1:** Biological PASS prediction spectrum computation.

S/N	Antiviral		Antibacterial		Antifungal		Antineoplastic	
	Pa	Pi	Pa	Pi	Pa	Pi	Pa	Pi
1	0.334	0.026	0.421	0.025	0.508	0.029	0.841	0.008
2	0.296	0.038	0.374	0.037	0.471	0.036	0.797	0.012
3	0.313	0.032	0.338	0.047	0.424	0.045	0.792	0.013
4	0.246	0.062	0.358	0.041	0.46	0.038	0.792	0.013
5	0.261	0.054	0.322	0.052	0.411	0.048	0.788	0.013
6	0.254	0.058	0.401	0.03	0.343	0.065	0.826	0.009
7	0.236	0.069	0.372	0.037	0.296	0.082	0.812	0.01
8	0.343		0.339	0.046	0.291	0.085	0.924	0.005
9	0.304	0.035	0.274	0.07	0.226	0.121	0.938	0.008

### Lipinski rule, pharmacokinetics, and drug-likeness

Having a lower and poor bioavailability is a big reason for potential drugs to fail and not reach the target site. Computing methodologies, such as the Lipinski rule, for measuring drug-likeness may reduce these concerns to an absolute minimum (Hosfield et al., 2021). Any bioactive molecule should follow the Lipinski rule to be a good and effective oral bioactive molecule (Santos et al., 2016) and provide a particular physiochemical or biological effectiveness. In pointing out and verifying the Lipinski rule on the reported molecules, it was seen that all the developed bioactive molecules actively accept the Lipinski rule, excluding ligand 7; but ligands 1, 5, 6, 8, and 9 have 1 violation of the Lipinski rule due to the hydrogen bond acceptor being not matched.

Secondly, the molecular weight level is measured at 318.24–530.48, while the bioavailability score is about 0.55 for all compounds, excluding ligands 7. This may happen due to the presence of the COOH functional group. Finally, all the bioactive molecules are poorly absorbed in the gastrointestinal tract (**Table 2**).

**Table 2:** Data on Lipinski rule, pharmacokinetics, and drug-likeness.

Ligand number	NBR	HBA	HBD	TPSA, Å²	Lipinski rule		M.W.	Bioavailability score	G.I. absorption
Result	Violation								
1	1	8	6	151.59	Yes	1	318.24	0.55	Low
2	3	8	5	140.59	Yes	0	394.33	0.55	Low
3	5	8	4	129.59	Yes	0	470.43	0.55	Low
4	4	9	5	149.82	Yes	0	424.36	0.55	Low
5	7	10	4	148.05	Yes	1	530.48	0.55	Low
6	3	10	6	177.89	Yes	1	362.24	0.11	Low
7	5	12	6	204.19	No	2	406.25	0.11	Low
8	2	9	6	166.61	Yes	1	333.25	0.55	Low
9	3	10	6	181.63	Yes	1	348.26	0.55	Low

TPSA, topological polar surface area; NBR, number of rotatable bonds; HBA, hydrogen bond acceptor; HBD, hydrogen bond donor; M.W., molecular weight; G.I. absorption, gastrointestinal absorption.

### Molecular docking and interaction analysis

Molecular docking assays were adopted in order to confirm the pharmacological and probable binding affinities (Kawsar et al., 2022) and demonstrate the affinity of pharmacological molecules 1–9 for breast cancer and lung cancer targeted proteins. The standard drug for breast cancer, epirubicin hydrochloride, and for lung cancer, carboplatin, were also studied, and docking experience was also adopted. Usually, -6.00 kcal/mol is a standard score for an effective biomolecule (Kawsar et al., 2022; Kumer et al., 2022), but we found and developed biomolecules that generated and banded with strong affinities to the targeted protein. In breast cancer protein PDB: 3HB5, the primary compound myricetin was reported at -9.5 kcal/mol, but, by adding or modifying the side chain, the docking score increases or decreases, and the maximum score obtained in the addition of the benzene ring is -10.4 and –10.3 kcal/mol in ligands 2 and 3, respectively, whereas the lowest score was -7.3 kcal/mol in ligand 9. Another breast cancer protein, PDB: 7KCD, had a maximum score of -9.0 and -8.8 kcal/mol in ligands 3 and 5.

Moreover, lung cancer (PDB 6G76) has been actively banned with a maximum docking score in ligands 2 and 3, whereas lung cancer (PDB 2ITO) has been significantly found at -10.1 and -9.6 in ligands 3 and 5, respectively. This suggested that this bioactive molecule could be an excellent anticancer agent for breast and lung cancer. In all four breast and lung cancer proteins, the newly developed biomolecules have better and potential effectiveness (**Table 3**).

**Table 3:** Binding affinity against breast and lung cancer protein.

Drug molecules number				
Breast cancer protein PDB: 3HB5	Breast cancer protein PDB: 7KCD	Lung cancer (PDB 6G76)	Lung cancer (PDB 2ITO)
Binding affinity(kcal/mol)	Binding affinity(kcal/mol)	Binding affinity(kcal/mol)	Binding affinity(kcal/mol)
01	-9.5	-8.3	-8.3	-8.8
02	-10.4	-7.8	-9.5	-9.0
03	-10.3	-9.0	-9.6	-10.1
04	-9.6	-8.4	-9.1	-9.0
05	-8.6	-8.8	-8.8	-9.6
06	-9.3	-7.3	-8.7	-8.6
07	-9.5	-6.8	-8.2	-8.2
08	-9.0	-8.5	-8.4	-8.7
09	-7.3	-8.1	-8.5	-8.6
Epirubicin hydrochloride	-8.3	-6.9	…	.
Carboplatin	—	—	-5.5	-5.4

### Protein–ligand interaction, molecular docking poses, and active site analysis

**Figure 6:**
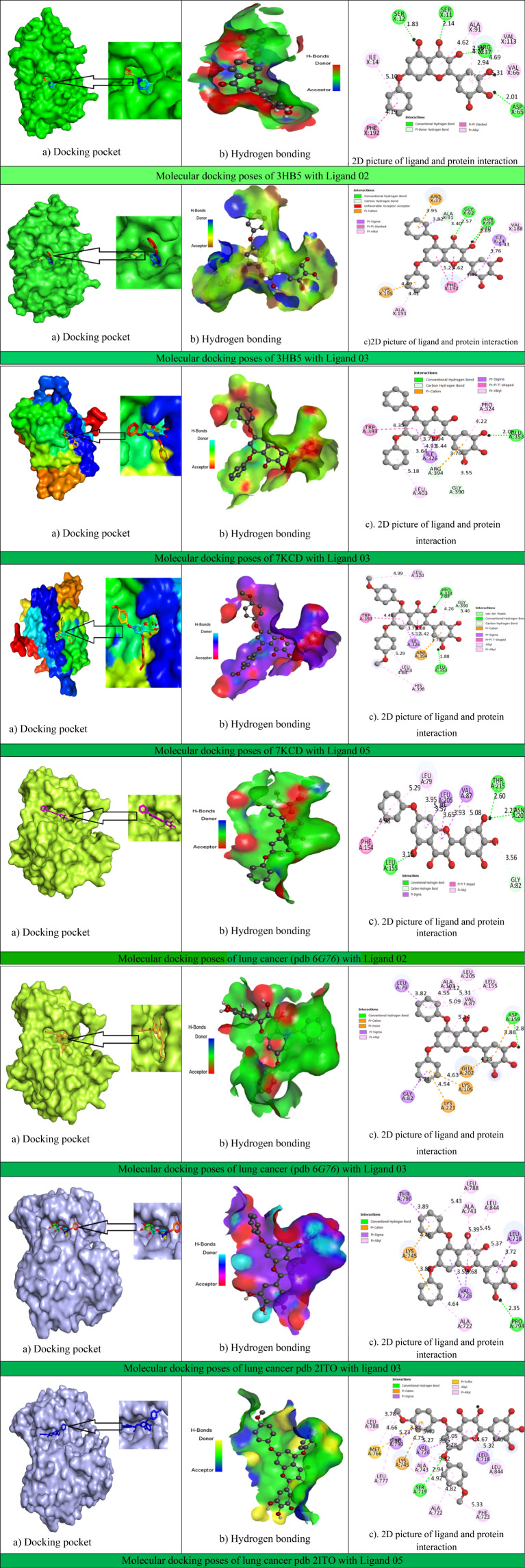
Docking interactions between the proposed compound and breast–lung cancer, hydrogen bonding, and 2D picture of the active sites.

**Figure 6** and **Supplementary Table S1** show that all myricetin-based bioactive substances established various hydrophilic and hydrophobic contacts and electrostatic interactions with breast cancer (PDB ID: 3hb5 and 7kcd) and lung cancer protein (PDB ID 6g76 and 2ito) chains. This active site has been analyzed by Biovia discovery studio 2020.

In ligand interaction with breast cancer protein PDB: 3hb5, the X: PHE192 active amino acid was present at a maximum of 14 times, and X: VAL188 was present 8 times, whereas the ligand interaction with breast cancer protein PDB: 7KCD has been shown [A: ALA350 (11 times) and A: LEU387 (9 times)] as the maximum active side.

In addition, lung cancer (PDB 6G76) targeted protein has been obtained, with A: VAL87 having the active side (18 times) with the maximum number of ligands, and the final one is lung cancer protein PDB 2ito, whose active site is A: VAL726 with a maximum number of around 23 times.

Thus, it is concluded that the active sides found against this targeted protein are valine-188, phenylalanine-192, leucine-387, alanine-350, valine-87, and valine-726, which have been displayed in most cases.

### ADMET prediction

ADMET is a critical step toward the invention of effective bioactive molecules since the drug may have constraints, such as poisonous or weak ADMET characteristics (Mazumdar et al., 2009). Therefore, the estimation of ADMET features is considered a crucial component in reducing the probability of possible problems arising after the invention and in the course of experimental or clinical therapies (Yun et al., 2007). The reported novel myricetin derivatives are predicted to have ADMET characteristics using pkCSM (Pires et al., 2015; Kumer et al., 2021) and SwissADME (Daina et al., 2017). Additionally, solubility in water is expressed as a logarithm (mol/L; insoluble <-10, weakly soluble <-6, slightly soluble <-4, soluble <-2, and very soluble <0) (Rout et al., 2022). The reported myricetin derivatives have Log S from -2.895 to -3.746, which fall in the -2 to -4 range. This means that the myricetin derivatives are soluble in a water medium. The results in **Table 4** show that none of the eight myricetin derivatives can pass through Caco-2’s permeability barrier and the blood–brain barrier (having high permeability would lead to a projected value greater than 0.9) (Khaldan et al., 2021). The VDss is expressed as log L/kg (low VDss < 0.71 L/kg or log <-0.15 and high if VDss < 2.81 L/kg or log >0.45). Based on these standard ranges, the VDss of the reported molecules presented a high distribution, while only ligands 1, 2, and 3 can inhibit the CYP450 1A2 inhibitor, and ligands 2 and 4 can only inhibit the CYP450 2C9 inhibitor. The total clearance rates are found from 0.312 to 0.831 ml/min/kg, of which the maximum value of 0.831 ml/min/kg of drug can be cleared from the body, and the minimum value is 0.312 ml/min/kg. Finally, no drug can be excreted through the renal OCT2 substrate (**Table 4**).

**Table 4:** Computation of ADME features.

S/N	Absorption		Distribution		Metabolism		Excretion	
Water solubility, log S	Caco-2 permeability (10^-6^ cm/s)	VDss (human) (log L/kg)	BBBpermeability	CYP450 1A2 inhibitor	CYP450 2C9 inhibitor	Total clearance (ml/min/kg)	Renal OCT2 substrate
01	-2.988	0.241	0.332	No	Yes	No	0.574	No
02	-3.746	0.409	-0.902	No	Yes	Yes	0.312	No
03	-3.304	0.352	-1.587	No	No	No	0.372	No
04	-2.904	0.512	-0.485	No	No	Yes	0.730	No
05	-2.895	0.592	-0.633	No	No	No	0.831	No
06	-3.006	0.356	-0.081	No	No	No	0.607	No
07	-2.982	0.658	-0.175	No	No	No	0.578	No
08	-3.05	0.465	0.101	No	Yes	No	0.670	No
09	-3.086	0.626	0.074	No	No	No	0.694	No
Epirubicin hydrochloride	-4.171	-0.139	-0.814	No	No	No	0.635	No
Carboplatin	-0.513	0.055	-0.791	No	No	No	0.419	

### Aquatic and non-aquatic toxicity

Aquatic and non-aquatic toxicity is another factor influencing drug safety and efficacy. The parameters for the prediction of aquatic and non-aquatic toxicity include AMES toxicity, maximum tolerated dose, oral rat acute toxicity, oral rat chronic toxicity, hepatotoxicity, and skin sensitization.

It was seen that almost all ligands were outside the AMES toxicity level, excluding ligands 1 and 7. The maximum tolerated dose level was 0.280–1.059280 mg/kg/day. A maximum of 1.059280 mg/kg/day should be administered in patients based on the score of the oral rat acute toxicity in the range 2.211–2.518 mol/kg and an oral rat chronic toxicity level of 3.167–4.446 mg/kg/day. Finally, no drug may produce hepatotoxicity or skin sensitization (**Table 5**).

**Table 5:** Aquatic and non-aquatic toxicity value prediction.

S/N	AMES toxicity	Maximum tolerated dose (human), mg/kg/day	Oral rat acute toxicity (LD50), (mol/kg)	Oral rat chronic toxicity (mg/kg/day)	Hepatotoxicity	Skin sensitization
01	Yes	0.997	2.358	3.728	No	No
02	No	0.563	2.419	3.167	No	No
03	No	0.280	2.774	3.619	No	No
04	No	0.480	2.383	4.133	No	No
05	No	0.379	2.518	4.446	No	No
06	No	0.979	2.211	3.936	No	No
07	Yes	0.804	2.44	4.229	No	No
08	No	1.053	2.452	3.871	No	No
09	No	1.059	2.468	3.915	No	No
Epirubicin hydrochloride	No	0.176	2.535	2.305	No	No
Carboplatin	No	1.11	1.992	2.847	No	No

## Conclusions

A series of myricetin derivative compounds were subjected to computational studies, such as molecular docking experiments, ADMET, drug-likeness, pass perdition, *etc.*, to develop novel, potent inhibitors against breast and lung cancer. This analysis allowed us to identify nine myricetin derivative compounds with a significant inhibitory action based on their binding affinities. The intermolecular docking score found was reported to be -10.4 and -10.3 kcal/mol in ligands 2 and 3, respectively, whereas the lowest score was -7.3 kcal/mol in ligand 9 against breast cancer protein PDB: 3hb5, and the targeted protein of breast cancer PDB: 7KCD obtained -9.0 and -8.8 kcal/mol docking scores in ligands 3 and 5, respectively (between the two targeted proteins, the most active molecule is ligand 2).

Furthermore, in contrast with lung cancer PDB 6G76, ligands 2 and 3 have gained more potency at -9.5 and -9.6 kcal/mol, respectively. Similarly, lung cancer (PDB 2ITO) has gained -10.1 and -9.6 in ligands 3 and 5 binding affinities. Therefore, the proposed bioactive myricetin derivatives and the crystal structure of breast and lung cancer showed that all the compounds have excellent binding energy and better stability in the active site of the experimental protein. It is concluded that myricetin derivatives have different types of active amino acid generated during the formation of drug protein complexes such as valine-188, phenylalanine-192, leucine-387, alanine-350, valine-87, and valine-726, and they have obtained a strong binding energy compared with the standard, outstanding pharmacokinetic features, adequate absorption, good metabolic transformation, and lower adverse effects. Thus, they have been proposed as viable breast and lung cancer inhibitors.

## Data availability statement

The original contributions presented in the study are included in the article/**Supplementary Material**. Further inquiries can be directed to the corresponding authors.

## Ethics statement

The reported studies did not involve human participants and human data.

## Author contributions

SA, AK, TE, and PW conceptualized and designed the manuscript as well as participated in drafting of the article and/or acquisition of data and/or analysis and interpretation of data. SA, AK, MR, RS, RKS, and FA prepared the figures and tables. TE, MP, MK, AI, PW, and BK wrote, edited, and revised the manuscript critically. TE, PW, and BK revised the final manuscript. All authors contributed to the article and approved the submitted version.

## Funding

This research was supported by Basic Science Research Program through the National Research Foundation of Korea (NRF) funded by the Ministry of Education (NRF-2020R1I1A2066868), the National Research Foundation of Korea (NRF) grant funded by the Korea government (MSIT) (No. 2020R1A5A2019413), a grant of the Korea Health Technology R&D Project through the Korea Health Industry Development Institute (KHIDI), funded by the Ministry of Health & Welfare, Republic of Korea (grant number : HF20C0116), and a grant of the Korea Health Technology R&D Project through the Korea Health Industry Development Institute (KHIDI), funded by the Ministry of Health & Welfare, Republic of Korea (grant number : HF20C0038). The authors would like to express their gratitude to King Khalid University, Saudi Arabia, for providing administrative and technical support.

## Acknowledgments

The authors would like to express their gratitude to King Khalid University, Saudi Arabia, for providing administrative and technical support.

## Conflict of interest

The authors declare that the research was conducted in the absence of any commercial or financial relationships that could be construed as a potential conflict of interest.

## Publisher’s note

All claims expressed in this article are solely those of the authors and do not necessarily represent those of their affiliated organizations, or those of the publisher, the editors and the reviewers. Any product that may be evaluated in this article, or claim that may be made by its manufacturer, is not guaranteed or endorsed by the publisher.
