# Supplementary material for: Development of new bioactive molecules to treat breast and lung cancer with natural myricetin and its derivatives: A computational and SAR approach
Source: Front Cell Infect Microbiol. 2022 Sep 27;12:952297. doi: 10.3389/fcimb.2022.952297 (PMC9551266; doi:10.3389/fcimb.2022.952297)
Supplement: Supplementary file 1 [file DataSheet_1.pdf]

Substitute Table 1: Breast cancer and Lung cancer protein- ligands interaction with amino acid (AA) residues and their bond distance

Breast cancer protein PDB: 3hb5

NO	Name	Distance	Category	Type
01	X: SER12	2.8869	Hydrogen Bond	Conventional Hydrogen Bond
	X: GLY15	1.91265	Hydrogen Bond	Conventional Hydrogen Bond
	X: TYR155	2.78419	Hydrogen Bond	Conventional Hydrogen Bond
	X: LYS159	2.32349	Hydrogen Bond	Conventional Hydrogen Bond
	X: GLY9:O	2.12232	Hydrogen Bond	Conventional Hydrogen Bond
	X: ASN90	2.28567	Hydrogen Bond	Conventional Hydrogen Bond
	X: GLY186	2.46244	Hydrogen Bond	Conventional Hydrogen Bond
	X: GLY141	2.41381	Hydrogen Bond	Conventional Hydrogen Bond
	X: SER142	3.02059	Hydrogen Bond	Carbon Hydrogen Bond
	X: PHE192	5.68639	Hydrophobic	Pi-Pi Stacked
	X: PHE192	4.87669	Hydrophobic	Pi-Pi Stacked
	X: PHE192	5.1997	Hydrophobic	Pi-Pi T-shaped
	X: CYS185	4.86022	Hydrophobic	Pi-Alkyl
	X: VAL188	5.11439	Hydrophobic	Pi-Alkyl
	X: ILE14	5.34241	Hydrophobic	Pi-Alkyl
02	X: SER11	2.14003	Hydrogen Bond	Conventional Hydrogen Bond
	X: ARG37	2.5608	Hydrogen Bond	Conventional Hydrogen Bond
	X:ASP65	2.01137	Hydrogen Bond	Conventional Hydrogen Bond
	X: SER12	1.83262	Hydrogen Bond	Conventional Hydrogen Bond
	X: ARG37	2.93798	Hydrogen Bond	Pi-Donor Hydrogen Bond
	X: PHE192	4.18829	Hydrophobic	Pi-Pi Stacked
	X: ALA91	4.62263	Hydrophobic	Pi-Alkyl
	X: ARG37	3.9088	Hydrophobic	Pi-Alkyl
	X: VAL66	5.30535	Hydrophobic	Pi-Alkyl
	X: ALA91	4.2148	Hydrophobic	Pi-Alkyl
	X: VAL113	4.68796	Hydrophobic	Pi-Alkyl
	X: ILE14	5.10256	Hydrophobic	Pi-Alkyl
03	X: GLY92	2.56668	Hydrogen Bond	Conventional Hydrogen Bond
	X: ASN90	2.39955	Hydrogen Bond	Conventional Hydrogen Bond
	X: ALA91	3.39898	Hydrogen Bond	Carbon Hydrogen Bond
	X: ARG37	3.9463	Electrostatic	Pi-Cation
	X: LYS195	4.48812	Electrostatic	Pi-Cation
	X: ILE14	3.76482	Hydrophobic	Pi-Sigma
	X: ALA91	3.81579	Hydrophobic	Pi-Sigma
	X: PHE192	5.25413	Hydrophobic	Pi-Pi Stacked
	X: PHE192	4.62454	Hydrophobic	Pi-Pi Stacked
	X: PHE192	4.45855	Hydrophobic	Pi-Pi Stacked
	X: VAL188	5.43193	Hydrophobic	Pi-Alkyl
	X: ARG37	4.63931	Hydrophobic	Pi-Alkyl
	X: ALA191	4.41295	Hydrophobic	Pi-Alkyl
	X: LYS195	4.1435	Hydrophobic	Pi-Alkyl
04	X: ARG37	2.79165	Hydrogen Bond	Conventional Hydrogen Bond
	X: ASN90	2.56205	Hydrogen Bond	Conventional Hydrogen Bond
	X:PRO187	3.55911	Hydrogen Bond	Carbon Hydrogen Bond
	X: PHE192	5.5671	Hydrophobic	Pi-Pi Stacked
	X: ILE14	5.25557	Hydrophobic	Pi-Alkyl
	X: VAL188	4.05897	Hydrophobic	Pi-Alkyl

05	X: SER12	2.51954	Hydrogen Bond	Conventional Hydrogen Bond
	X: ARG37	2.41815	Hydrogen Bond	Conventional Hydrogen Bond
	X: ASN90	2.3843	Hydrogen Bond	Conventional Hydrogen Bond
	X: LYS195	2.41825	Hydrogen Bond	Conventional Hydrogen Bond
	X: GLY92	1.78895	Hydrogen Bond	Conventional Hydrogen Bond
	X: LEU93	3.52532	Hydrophobic	Pi-Sigma
	X: LYS195	3.95299	Hydrophobic	Pi-Sigma
	X: PHE192	5.15436	Hydrophobic	Pi-Pi Stacked
	X: LEU93	4.27931	Hydrophobic	Alkyl
	X: LYS195	5.11293	Hydrophobic	Pi-Alkyl
	X: ALA191	4.20915	Hydrophobic	Pi-Alkyl
06	X: GLY15	1.97481	Hydrogen Bond	Conventional Hydrogen Bond
	X: LYS159	1.97286	Hydrogen Bond	Conventional Hydrogen Bond
	X: GLY9	2.17484	Hydrogen Bond	Conventional Hydrogen Bond
	X: SER12	2.31825	Hydrogen Bond	Conventional Hydrogen Bond
	X: SER12	2.98592	Hydrogen Bond	Conventional Hydrogen Bond
	X: GLY92	2.07998	Hydrogen Bond	Conventional Hydrogen Bond
	X: PHE192	5.5889	Hydrophobic	Pi-Pi Stacked
	X: PHE192	5.03579	Hydrophobic	Pi-Pi Stacked
	X: CYS185	4.95712	Hydrophobic	Pi-Alkyl
	X: VAL188	5.43934	Hydrophobic	Pi-Alkyl
07	X: SER12	2.24805	Hydrogen Bond	Conventional Hydrogen Bond
	X: LYS159	2.36817	Hydrogen Bond	Conventional Hydrogen Bond
	X: VAL188	2.21093	Hydrogen Bond	Conventional Hydrogen Bond
	X: SER11	2.18251	Hydrogen Bond	Conventional Hydrogen Bond
	X: GLY141	2.06756	Hydrogen Bond	Conventional Hydrogen Bond
	X: ILE14	3.90308	Hydrophobic	Pi-Sigma
	X: PHE192	4.3151	Hydrophobic	Pi-Pi Stacked
	X: PHE192	4.24573	Hydrophobic	Pi-Pi Stacked
	X: VAL188	5.40368	Hydrophobic	Pi-Alkyl
08	X: ARG37	2.96447	Hydrogen Bond	Conventional Hydrogen Bond
	X: GLY9	2.21819	Hydrogen Bond	Conventional Hydrogen Bond
	X: VAL188	1.81069	Hydrogen Bond	Conventional Hydrogen Bond
	X: ILE14	3.96656	Hydrophobic	Pi-Sigma
	X: PHE192	4.26213	Hydrophobic	Pi-Pi Stacked
	X: PHE192	4.14844	Hydrophobic	Pi-Pi Stacked
	X: VAL188	5.3021	Hydrophobic	Pi-Alkyl
09	X: HIS280	2.82772	Hydrogen Bond	Conventional Hydrogen Bond
	X:ASP153	1.97999	Hydrogen Bond	Conventional Hydrogen Bond
	X:ASP208	2.46032	Hydrogen Bond	Conventional Hydrogen Bond
	X: PHE151	5.96928	Hydrophobic	Pi-Pi Stacked
	X: PHE151	4.22366	Hydrophobic	Pi-Pi Stacked
	X: ARG214	4.40212	Hydrophobic	Pi-Alkyl
	X: ARG214	4.3097	Hydrophobic	Pi-Alkyl
**Breast cancer protein *PDB: 7KCD***				
**NO**	Name	Distance	Category	Type
**01**	A:ASP351	2.75457	Hydrogen Bond	Conventional Hydrogen Bond
	A: THR347	2.15531	Hydrogen Bond	Conventional Hydrogen Bond
	A: LEU387	2.10261	Hydrogen Bond	Conventional Hydrogen Bond
	A: LEU525	3.85668	Hydrophobic	Pi-Sigma

	A: LEU387	4.8022	Hydrophobic	Pi-Alkyl
	A: MET388	5.08293	Hydrophobic	Pi-Alkyl
	A: ALA350	4.80767	Hydrophobic	Pi-Alkyl
	A: LEU384	4.85834	Hydrophobic	Pi-Alkyl
	A: ALA350	3.85329	Hydrophobic	Pi-Alkyl
**02**	A: LEU544	2.30601	Hydrogen Bond	Conventional Hydrogen Bond
	A: VAL368	2.19666	Hydrogen Bond	Conventional Hydrogen Bond
	A: LEU372	3.71162	Hydrogen Bond	Pi-Donor Hydrogen Bond
	A: LEU372	3.74746	Hydrogen Bond	Pi-Donor Hydrogen Bond
	A: LEU372	3.60708	Hydrophobic	Pi-Sigma
**03**	A: GLU353	2.04588	Hydrogen Bond	Conventional Hydrogen Bond
	A: GLY390	3.55327	Hydrogen Bond	Carbon Hydrogen Bond
	A: ARG394	3.63945	Hydrogen Bond	Carbon Hydrogen Bond
	A: ARG394	3.76098	Electrostatic	Pi-Cation
	A: ILE326	3.70917	Hydrophobic	Pi-Sigma
	A: ILE326	3.94055	Hydrophobic	Pi-Sigma
	A: TRP393	4.34793	Hydrophobic	Pi-Pi T-shaped
	A: ARG394	4.93052	Hydrophobic	Pi-Alkyl
	A: ARG394	5.43562	Hydrophobic	Pi-Alkyl
	A:PRO324	4.21528	Hydrophobic	Pi-Alkyl
	A: LEU403	5.18339	Hydrophobic	Pi-Alkyl
**04**	A: THR347	3.05447	Hydrogen Bond	Conventional Hydrogen Bond
	A: LEU387	2.28918	Hydrogen Bond	Conventional Hydrogen Bond
	A: THR347	2.34665	Hydrogen Bond	Conventional Hydrogen Bond
	A:ASP351	3.67852	Hydrogen Bond	Carbon Hydrogen Bond
	A:ASP351	4.77851	Electrostatic	Pi-Anion
	A:ASP351	3.23671	Electrostatic	Pi-Anion
	A: LEU525	3.60225	Hydrophobic	Pi-Sigma
	A: LEU525	3.94914	Hydrophobic	Pi-Sigma
	A: MET343	5.15624	Other	Pi-Sulfur
	A: TRP383	5.85913	Hydrophobic	Pi-Pi T-shaped
	A: LEU346	4.65459	Hydrophobic	Amide-Pi Stacked
	A: LEU354	4.6557	Hydrophobic	Alkyl
	A: LEU539	5.1771	Hydrophobic	Alkyl
	A: ALA350	4.30352	Hydrophobic	Pi-Alkyl
	A: ALA350	3.87551	Hydrophobic	Pi-Alkyl
	A: LEU525	4.89176	Hydrophobic	Pi-Alkyl
	A: ALA350	4.93499	Hydrophobic	Pi-Alkyl
	A: LEU387	4.57844	Hydrophobic	Pi-Alkyl
	A: LEU354	5.0877	Hydrophobic	Pi-Alkyl
	A:PRO535	4.36701	Hydrophobic	Pi-Alkyl
**05**	A: GLU353	1.88167	Hydrogen Bond	Conventional Hydrogen Bond
	A:PRO324	2.0217	Hydrogen Bond	Conventional Hydrogen Bond
	A: GLY390	3.45788	Hydrogen Bond	Carbon Hydrogen Bond
	A: ARG394	3.70485	Electrostatic	Pi-Cation
	A: ILE326	3.76818	Hydrophobic	Pi-Sigma
	A: ILE326	3.88401	Hydrophobic	Pi-Sigma
	A: TRP393	4.39655	Hydrophobic	Pi-Pi T-shaped
	A: LEU320	4.99451	Hydrophobic	Alkyl
	A: HIS398	4.64334	Hydrophobic	Pi-Alkyl
	A: ARG394	5.11787	Hydrophobic	Pi-Alkyl

	A: ARG394<br>A:PRO324<br>A: LEU403	5.42499<br>4.2637<br>5.29357	Hydrophobic<br>Hydrophobic<br>Hydrophobic	Pi-Alkyl<br>Pi-Alkyl<br>Pi-Alkyl
**06**	A: THR483<br>A: THR483<br>A: SER512<br>A: ARG515<br>A: ARG515<br>A: LEU479<br>A: GLU385<br>A: LEU511<br>A: LEU508<br>A: LEU479	2.9863<br>3.08328<br>3.23264<br>3.3903<br>3.39851<br>2.73936<br>2.08951<br>4.63501<br>4.27808<br>4.89247	Hydrogen Bond<br>Hydrogen Bond<br>Hydrogen Bond<br>Hydrogen Bond<br>Hydrogen Bond<br>Hydrogen Bond<br>Hydrogen Bond<br>Hydrophobic<br>Hydrophobic<br>Hydrophobic	Conventional Hydrogen Bond<br>Conventional Hydrogen Bond<br>Conventional Hydrogen Bond<br>Conventional Hydrogen Bond<br>Conventional Hydrogen Bond<br>Conventional Hydrogen Bond<br>Conventional Hydrogen Bond<br>Pi-Alkyl<br>Pi-Alkyl<br>Pi-Alkyl
**07**	A: THR483<br>A: LEU508<br>A: SER512<br>A: ASN455<br>A: LEU508<br>A: LEU511<br>A: LEU508<br>A: LEU511	3.20457<br>2.2211<br>3.01257<br>3.3042<br>3.72075<br>4.16232<br>4.67746<br>4.6302	Hydrogen Bond<br>Hydrogen Bond<br>Hydrogen Bond<br>Hydrogen Bond<br>Hydrophobic<br>Hydrophobic<br>Hydrophobic<br>Hydrophobic	Conventional Hydrogen Bond<br>Conventional Hydrogen Bond<br>Conventional Hydrogen Bond<br>Pi-Donor Hydrogen Bond<br>Pi-Sigma<br>Amide-Pi Stacked<br>Pi-Alkyl<br>Pi-Alkyl
**08**	A: THR347<br>A: GLU353<br>A: GLU353<br>A: LEU525<br>A: LEU525<br>A: MET343<br>A: ALA350<br>A: LEU387<br>A: ALA350<br>A: LEU384<br>A: LEU387<br>A: ALA350	22.70188<br>2.58872<br>2.98036<br>3.6698<br>3.90762<br>5.8867<br>5.00459<br>4.64742<br>4.48145<br>5.11998<br>5.40094<br>4.16032	Hydrogen Bond<br>Hydrogen Bond<br>Hydrogen Bond<br>Hydrophobic<br>Hydrophobic<br>Other<br>Hydrophobic<br>Hydrophobic<br>Hydrophobic<br>Hydrophobic<br>Hydrophobic<br>Hydrophobic	Conventional Hydrogen Bond<br>Conventional Hydrogen Bond<br>Conventional Hydrogen Bond<br>Pi-Sigma<br>Pi-Sigma<br>Pi-Sulfur<br>Pi-Alkyl<br>Pi-Alkyl<br>Pi-Alkyl<br>Pi-Alkyl<br>Pi-Alkyl<br>Pi-Alkyl
**09**	A: THR347<br>A: GLU353<br>A: LEU387<br>A: LEU525<br>A: LEU525<br>A: MET343<br>A: ALA350<br>A: LEU387<br>A: ALA350<br>A: LEU384<br>A: LEU387<br>A: ALA350	2.71437<br>1.99299<br>2.60372<br>3.71987<br>3.96573<br>5.94581<br>4.89642<br>4.69613<br>4.40065<br>5.21858<br>5.47373<br>4.16305	Hydrogen Bond<br>Hydrogen Bond<br>Hydrogen Bond<br>Hydrophobic<br>Hydrophobic<br>Other<br>Hydrophobic<br>Hydrophobic<br>Hydrophobic<br>Hydrophobic<br>Hydrophobic<br>Hydrophobic	Conventional Hydrogen Bond<br>Conventional Hydrogen Bond<br>Conventional Hydrogen Bond<br>Pi-Sigma<br>Pi-Sigma<br>Pi-Sulfur<br>Pi-Alkyl<br>Pi-Alkyl<br>Pi-Alkyl<br>Pi-Alkyl<br>Pi-Alkyl<br>Pi-Alkyl
		Lung cancer (pdb 6G76)		
**01**	A: PHE84<br>A: GLY85<br>A: SER83<br>A:ASP153<br>A: LYS105	3.1657<br>2.11124<br>2.83109<br>2.25157<br>3.19287	Hydrogen Bond<br>Hydrogen Bond<br>Hydrogen Bond<br>Hydrogen Bond<br>Hydrogen Bond	Conventional Hydrogen Bond<br>Conventional Hydrogen Bond<br>Conventional Hydrogen Bond<br>Conventional Hydrogen Bond<br>Carbon Hydrogen Bond

	A: LYS105	4.13288	Electrostatic	Pi-Donor Hydrogen Bond
	A: LYS221	4.7286	Electrostatic	Pi-Cation
	A: VAL87	3.76129	Hydrophobic	Pi-Sigma
	A: VAL87	3.30318	Hydrophobic	Pi-Sigma
	A: THR215	3.95753	Hydrophobic	Pi-Sigma
	A: ALA103	4.71776	Hydrophobic	Pi-Alkyl
**02**	A: LEU155	3.16202	Hydrogen Bond	Conventional Hydrogen Bond
	A: ASN203	2.26666	Hydrogen Bond	Conventional Hydrogen Bond
	A: THR215	2.60179	Hydrogen Bond	Conventional Hydrogen Bond
	A: GLY82	3.56223	Hydrogen Bond	Carbon Hydrogen Bond
	A: VAL87	3.92921	Hydrophobic	Pi-Sigma
	A: LEU205	3.56854	Hydrophobic	Pi-Sigma
	A: LEU205	3.64559	Hydrophobic	Pi-Sigma
	A: PHE154	4.98376	Hydrophobic	Pi-Pi T-shaped
	A: LEU79	3.94521	Hydrophobic	Pi-Alkyl
	A: LEU79	5.30426	Hydrophobic	Pi-Alkyl
	A: VAL87	5.07923	Hydrophobic	Pi-Alkyl
	A: LEU79	5.28737	Hydrophobic	Pi-Alkyl
**03**	A:ASP159	2.85163	Hydrogen Bond	Conventional Hydrogen Bond
	A: LYS105	4.63332	Electrostatic	Pi-Cation
	A: LYS221	4.53578	Electrostatic	Pi-Cation
	A:ASP159	3.85605	Electrostatic	Pi-Anion
	A: GLU202	4.27701	Electrostatic	Pi-Anion
	A: LEU79	3.81991	Hydrophobic	Pi-Sigma
	A: GLY82	3.74225	Hydrophobic	Pi-Sigma
	A: GLU202	3.45481	Hydrophobic	Pi-Sigma
	A: VAL87	5.13715	Hydrophobic	Pi-Alkyl
	A: VAL87	5.09242	Hydrophobic	Pi-Alkyl
	A: ALA103	4.54828	Hydrophobic	Pi-Alkyl
	A: LEU155	5.30738	Hydrophobic	Pi-Alkyl
	A: LEU205	5.12137	Hydrophobic	Pi-Alkyl
**04**	A: THR215	3.23504	Hydrogen Bond	Conventional Hydrogen Bond
	A: ASN203	2.58211	Hydrogen Bond	Conventional Hydrogen Bond
	A: VAL87	3.92944	Hydrophobic	Pi-Sigma
	A: LEU205	3.55224	Hydrophobic	Pi-Sigma
	A: LEU205	3.62717	Hydrophobic	Pi-Sigma
	A: PHE154	5.00117	Hydrophobic	Pi-Pi T-shaped
	A: PHE154	5.48709	Hydrophobic	Pi-Alkyl
	A: LEU79	3.95974	Hydrophobic	Pi-Alkyl
	A: LEU79	5.32427	Hydrophobic	Pi-Alkyl
	A: VAL87	5.02367	Hydrophobic	Pi-Alkyl
	A: LEU79	5.28888	Hydrophobic	Pi-Alkyl
**05**	A:ASP159	1.86726	Hydrogen Bond	Conventional Hydrogen Bond
	A: GLY82	3.27292	Hydrogen Bond	Carbon Hydrogen Bond
	A: LYS200:	4.02889	Electrostatic	Pi-Donor Hydrogen Bond
	A:ASP159	3.56416	Electrostatic	Pi-Anion
	A: GLU202	4.77157	Electrostatic	Pi-Anion
	A: VAL87	3.56331	Hydrophobic	Pi-Sigma
	A: VAL136	3.44668	Hydrophobic	Alkyl
	A: LEU152	3.92312	Hydrophobic	Alkyl
	A: PHE84	5.37776	Hydrophobic	Pi-Alkyl

	A: ALA103	4.8493	Hydrophobic	Pi-Alkyl
	A: LEU205	5.44698	Hydrophobic	Pi-Alkyl
**06**	A: ASN203	2.17307	Hydrogen Bond	Conventional Hydrogen Bond
	A: THR215	2.91911	Hydrogen Bond	Conventional Hydrogen Bond
	A: LEU155	2.07056	Hydrogen Bond	Conventional Hydrogen Bond
	A:ASP153	2.9682	Hydrogen Bond	Conventional Hydrogen Bond
	A: LYS105	3.5233	Hydrogen Bond	Carbon Hydrogen Bond
	A: LEU79:	3.89725	Hydrophobic	Pi-Sigma
	A: VAL87	3.88759	Hydrophobic	Pi-Sigma
	A: LEU205	3.66211	Hydrophobic	Pi-Sigma
	A: LEU205	4.99258	Hydrophobic	Pi-Alkyl
	A: LEU79	5.33852	Hydrophobic	Pi-Alkyl
	A: VAL87	5.01734	Hydrophobic	Pi-Alkyl
**07**	A: THR215	2.99313	Hydrogen Bond	Conventional Hydrogen Bond
	A:ASP159	2.89012	Hydrogen Bond	Conventional Hydrogen Bond
	A:ASP153	2.17785	Hydrogen Bond	Conventional Hydrogen Bond
	A: GLU202	2.45649	Hydrogen Bond	Conventional Hydrogen Bond
	A: ASN203	2.44713	Hydrogen Bond	Conventional Hydrogen Bond
	A: VAL87	3.83759	Hydrophobic	Pi-Sigma
	A: VAL87	3.88822	Hydrophobic	Pi-Sigma
	A: LEU205	5.15742	Hydrophobic	Pi-Alkyl
	A: LEU79	4.43634	Hydrophobic	Pi-Alkyl
	A: VAL87	4.94804	Hydrophobic	Pi-Alkyl
	A: LEU205	4.70665	Hydrophobic	Pi-Alkyl
	A: LEU79	5.29578	Hydrophobic	Pi-Alkyl
**08**	A: GLY85	1.77207	Hydrogen Bond	Conventional Hydrogen Bond
	A: SER83	2.60724	Hydrogen Bond	Conventional Hydrogen Bond
	A:ASP153	2.74307	Hydrogen Bond	Conventional Hydrogen Bond
	A:ASP153	2.60254	Hydrogen Bond	Conventional Hydrogen Bond
	A: GLY80	3.33366	Hydrogen Bond	Carbon Hydrogen Bond
	A: LYS105	4.66541	Electrostatic	Pi-Cation
	A: GLY82	3.60372	Hydrophobic	Pi-Sigma
	A: VAL87	3.79782	Hydrophobic	Pi-Sigma
	A: VAL87	3.52747	Hydrophobic	Pi-Sigma
	A: LEU205	3.77119	Hydrophobic	Pi-Sigma
	A: LEU79	5.28214	Hydrophobic	Pi-Alkyl
**09**	A: LEU155	3.09717	Hydrogen Bond	Conventional Hydrogen Bond
	A: LEU155	1.78552	Hydrogen Bond	Conventional Hydrogen Bond
	A:ASP153	2.05123	Hydrogen Bond	Conventional Hydrogen Bond
	A: GLY82	3.46035	Hydrogen Bond	Carbon Hydrogen Bond
	A: LEU79	3.91705	Hydrophobic	Pi-Sigma
	A: LEU205	3.71659	Hydrophobic	Pi-Sigma
	A: VAL87	5.40975	Hydrophobic	Pi-Alkyl
	A: LEU205	4.97536	Hydrophobic	Pi-Alkyl
	A: LEU79	5.43666	Hydrophobic	Pi-Alkyl
	A: VAL87	4.13947	Hydrophobic	Pi-Alkyl
Lung cancer (pdb 2ito)				
**01**	A: MET793	2.1329	Hydrogen Bond	Conventional Hydrogen Bond
	A: GLU762	2.72847	Hydrogen Bond	Conventional Hydrogen Bond
	A: LEU718	2.24386	Hydrogen Bond	Conventional Hydrogen Bond

	A: MET793	2.52037	Hydrogen Bond	Conventional Hydrogen Bond
	A: LYS745	4.60804	Electrostatic	Pi-Cation
	A: LEU718	3.85523	Hydrophobic	Pi-Sigma
	A: VAL726	3.99797	Hydrophobic	Pi-Sigma
	A: VAL726	4.96799	Hydrophobic	Pi-Alkyl
	A: ALA743	4.28055	Hydrophobic	Pi-Alkyl
	A: LEU844	4.84423	Hydrophobic	Pi-Alkyl
	A: VAL726	5.34283	Hydrophobic	Pi-Alkyl
	A: LYS745	5.05033	Hydrophobic	Pi-Alkyl
**02**	A: LYS745	2.39927	Hydrogen Bond	Conventional Hydrogen Bond
	A: MET793	1.82392	Hydrogen Bond	Conventional Hydrogen Bond
	A: GLU762	2.91125	Hydrogen Bond	Conventional Hydrogen Bond
	A: LEU718	3.92186	Hydrophobic	Pi-Sigma
	A: VAL726	5.07982	Hydrophobic	Pi-Alkyl
	A: ALA743	4.41958	Hydrophobic	Pi-Alkyl
	A: LEU844	4.64356	Hydrophobic	Pi-Alkyl
	A: VAL726	5.4733	Hydrophobic	Pi-Alkyl
	A: LEU844	5.40414	Hydrophobic	Pi-Alkyl
**03**	A:PRO794	2.35114	Hydrogen Bond	Conventional Hydrogen Bond
	A: LYS745	4.45803	Electrostatic	Pi-Cation
	A: LYS745	3.88455	Electrostatic	Pi-Cation
	A: LEU718	3.71589	Hydrophobic	Pi-Sigma
	A: VAL726	3.6825	Hydrophobic	Pi-Sigma
	A: VAL726	3.51512	Hydrophobic	Pi-Sigma
	A: LYS745	3.90609	Hydrophobic	Pi-Sigma
	A: THR790	3.89014	Hydrophobic	Pi-Sigma
	A: LEU718	5.36572	Hydrophobic	Pi-Alkyl
	A: ALA743	5.39267	Hydrophobic	Pi-Alkyl
	A: LEU844	5.44793	Hydrophobic	Pi-Alkyl
	A: LYS745	4.20463	Hydrophobic	Pi-Alkyl
	A: LEU788	5.42954	Hydrophobic	Pi-Alkyl
	A: ALA722	4.63766	Hydrophobic	Pi-Alkyl
	A: LYS745	5.11112	Hydrophobic	Pi-Alkyl
**04**	A: LYS745	1.87488	Hydrogen Bond	Conventional Hydrogen Bond
	A: MET793	1.82943	Hydrogen Bond	Conventional Hydrogen Bond
	A:ASP855	2.42451	Hydrogen Bond	Conventional Hydrogen Bond
	A: GLU762	2.61474	Hydrogen Bond	Conventional Hydrogen Bond
	A:ASP800	3.98339	Electrostatic	Pi-Anion
	A: LEU718	3.9019	Hydrophobic	Pi-Sigma
	A: VAL726	5.0888	Hydrophobic	Pi-Alkyl
	A: ALA743	4.44708	Hydrophobic	Pi-Alkyl
	A: LEU844	4.64805	Hydrophobic	Pi-Alkyl
	A: VAL726	5.4733	Hydrophobic	Pi-Alkyl
	A: LEU844	5.38366	Hydrophobic	Pi-Alkyl
**05**	A: SER719	2.94161	Hydrogen Bond	Conventional Hydrogen Bond
	A: LYS745	4.74778	Electrostatic	Pi-Cation
	A: LEU718	3.40472	Hydrophobic	Pi-Sigma
	A: VAL726	3.95497	Hydrophobic	Pi-Sigma
	A: THR790	3.82424	Hydrophobic	Pi-Sigma
	A: MET766	5.22935	Other	Pi-Sulfur
	A: LYS745	4.91804	Hydrophobic	Alkyl

	A: MET766	4.66354	Hydrophobic	Alkyl
	A: LEU777	5.30361	Hydrophobic	Alkyl
	A: LEU788	3.77896	Hydrophobic	Alkyl
	A: PHE723	5.33026	Hydrophobic	Pi-Alkyl
	A: LEU718	4.67338	Hydrophobic	Pi-Alkyl
	A: VAL726	5.04948	Hydrophobic	Pi-Alkyl
	A: ALA743	5.27792	Hydrophobic	Pi-Alkyl
	A: LEU844	5.31909	Hydrophobic	Pi-Alkyl
	A: ALA722	4.82227	Hydrophobic	Pi-Alkyl
	A: VAL726	5.32207	Hydrophobic	Pi-Alkyl
	A: VAL726	5.39553	Hydrophobic	Pi-Alkyl
	A: ALA743	5.26902	Hydrophobic	Pi-Alkyl
	A: LYS745	4.84464	Hydrophobic	Pi-Alkyl
**06**	A: MET793	2.13223	Hydrogen Bond	Conventional Hydrogen Bond
	A: GLU762	2.72099	Hydrogen Bond	Conventional Hydrogen Bond
	A: LEU718	2.34052	Hydrogen Bond	Conventional Hydrogen Bond
	A: SER719	2.4245	Hydrogen Bond	Conventional Hydrogen Bond
	A: MET793	2.79142	Hydrogen Bond	Conventional Hydrogen Bond
	A: LYS745	4.55197	Electrostatic	Pi-Cation
	A: LEU718	3.92621	Hydrophobic	Pi-Sigma
	A: VAL726	3.99321	Hydrophobic	Pi-Sigma
	A: VAL726	4.94766	Hydrophobic	Pi-Alkyl
	A: ALA743	4.26375	Hydrophobic	Pi-Alkyl
	A: LEU844	4.82315	Hydrophobic	Pi-Alkyl
	A: VAL726	5.41261	Hydrophobic	Pi-Alkyl
	A: LYS745	5.01033	Hydrophobic	Pi-Alkyl
**07**	A: LYS745	2.08022	Hydrogen Bond	Conventional Hydrogen Bond
	A: LEU718	2.52462	Hydrogen Bond	Conventional Hydrogen Bond
	A: LEU718	3.85391	Hydrophobic	Pi-Sigma
	A: LEU718	3.92029	Hydrophobic	Pi-Sigma
	A: VAL726	3.71625	Hydrophobic	Pi-Sigma
	A: VAL726:	3.79428	Hydrophobic	Pi-Sigma
	A: LYS745	5.35734	Hydrophobic	Pi-Alkyl
	A: ALA743	4.60549	Hydrophobic	Pi-Alkyl
	A: LEU844	5.01722	Hydrophobic	Pi-Alkyl
**08**	A: MET793	1.83763	Hydrogen Bond	Conventional Hydrogen Bond
	A:ASP855	2.38144	Hydrogen Bond	Conventional Hydrogen Bond
	A:ASP855	2.32107	Hydrogen Bond	Conventional Hydrogen Bond
	A: MET793	2.03223	Hydrogen Bond	Conventional Hydrogen Bond
	A: LEU718	3.87085	Hydrophobic	Pi-Sigma
	A: VAL726	5.08357	Hydrophobic	Pi-Alkyl
	A: ALA743	4.47647	Hydrophobic	Pi-Alkyl
	A: LEU844	4.65217	Hydrophobic	Pi-Alkyl
	A: VAL726	5.44703	Hydrophobic	Pi-Alkyl
	A: LEU844	5.39006	Hydrophobic	Pi-Alkyl
**09**	A: MET793	2.09868	Hydrogen Bond	Conventional Hydrogen Bond
	A:ASP855	2.4289	Hydrogen Bond	Conventional Hydrogen Bond
	A: GLU762	2.58759	Hydrogen Bond	Conventional Hydrogen Bond
	A: MET793	2.5626	Hydrogen Bond	Conventional Hydrogen Bond
	A:PRO794	2.7362	Hydrogen Bond	Conventional Hydrogen Bond
	A:PRO794	2.68529	Hydrogen Bond	Conventional Hydrogen Bond

	A: LYS745	4.5742	Electrostatic	Pi-Cation
	A: LEU718	3.90325	Hydrophobic	Pi-Sigma
	A: VAL726	3.98695	Hydrophobic	Pi-Sigma
	A: VAL726	4.94704	Hydrophobic	Pi-Alkyl
	A: ALA743	4.23255	Hydrophobic	Pi-Alkyl
	A: LEU844	4.84207	Hydrophobic	Pi-Alkyl
	A: VAL726	5.39768	Hydrophobic	Pi-Alkyl
	A: LYS745	5.03107	Hydrophobic	Pi-Alkyl

[Note: TRP = Tryptophan, ASP = Aspartic acid, GLU = Glutamic acid, LEU = Leucine, THR = Threonine, ASN = Asparagine, GLN = Glutamine, PHE = Phenylalanine, ILE = Isoleucine, ARG = Arginine, VAL = Valine, SER = Serine, PRO = Proline, GLY = Glycine, HIS = Histidine, LYS = Lysine, TRP = Tryptophan, CYS = Cysteine, MET = Methionine.]
